# HOMA, BMI, and Serum Leptin Levels Variations during Antiviral Treatment Suggest Virus-Related Insulin Resistance in Noncirrhotic, Nonobese, and Nondiabetic Chronic Hepatitis C Genotype 1 Patients

**DOI:** 10.1155/2015/975695

**Published:** 2015-03-03

**Authors:** Alessandro Grasso, Federica Malfatti, Gabriella Andraghetti, Simona Marenco, Chiara Mazzucchelli, Sara Labanca, Renzo Cordera, Roberto Testa, Antonino Picciotto

**Affiliations:** ^1^Gastroenterology Unit, San Paolo Hospital, Via Genova 38, 17100 Savona, Italy; ^2^Department of Endocrinology, University of Genoa, Viale Benedetto XV 6, 16132 Genoa, Italy; ^3^Gastroenterology Unit, Department of Internal Medicine, University of Genoa, Viale Benedetto XV 6, 16132 Genoa, Italy

## Abstract

*Objective*. To investigate the relationship between insulin resistance and viral load decay in nondiabetic and noncirrhotic genotype 1 chronic HCV patients during peginterferon and ribavirin treatment and the possible influence of BMI and leptin as metabolic confounders. *Methods*. 75 consecutive noncirrhotic, nonobese, and nondiabetic patients with genotype 1 chronic hepatitis C treated with peginterferon alpha 2a plus ribavirin were evaluated. HOMA-IR, serum leptin, and BMI were measured in all patients at baseline and at weeks 12 and 48, whereas viral load was measured at the same time points and then 24 weeks after the end of treatment. *Results*. HOMA-IR was significantly associated with both BMI and leptin at baseline. During peginterferon plus ribavirin treatment, there was a significant reduction of HOMA-IR at weeks 12 and 48 from baseline (*P* = 0.033 and 0.048, resp.) in patients who achieved an early viral load decay (EVR), a trend not observed in patients who not achieved EVR. No variations during treatment were observed regarding BMI and leptin irrespective of EVR. *Conclusion*. The early reduction of HOMA-IR but not of BMI and leptin during antiviral treatment in noncirrhotic, chronic hepatitis C genotype 1 patients who achieved EVR suggests a viral genesis of insulin resistance in patients with nonmetabolic phenotype.

## 1. Introduction

The standard treatment of genotype 1 chronic hepatitis C with pegylated-interferon plus ribavirin is associated with a 40–50% rate of sustained viral response (SVR) [1–3].

A number of pretreatment variables related to the virus (hepatitis C virus genotypes 1 and 4, high viral load) and to the host (IL-28 polymorphism, age, overweight, and metabolic factors), as well as on-treatment variables (4-week negativization of viral load) have been demonstrated to affect the response to anti-viral therapy in patients with chronic hepatitis C [4–7].

Among host factors, overweight and insulin resistance are the most commonly studied since they are a possible target of intervention. One of the open issues today is the mutual influence of insulin resistance and viral load variations during antiviral treatment.

The relationship between insulin resistance and hepatitis C virus (HCV) is complex and still not completely elucidated. HCV genotype 1 is able to directly induce insulin resistance [8] by interfering with insulin signaling on hepatocytes [9]. Insulin resistance is a profibrogenetic stimulus; thus patients with high insulin resistance show more advanced liver fibrosis than patients with low insulin resistance [10].

There is robust evidence that antiviral treatment influences insulin resistance. Patients with SVR showed a significant reduction of insulin resistance from baseline to the end of follow-up compared with patients who did not achieve SVR [11, 12]. These data support the hypothesis that HCV may induce insulin resistance.

On the other hand, insulin resistance can influence antiviral response. Patients with high insulin resistance have a slower decay of HCV viral load than patients with low insulin resistance already in the early phase of treatment, suggesting that hyperinsulinaemia reduces the cellular response to pegylated-interferon [13]. Furthermore, lower SVR has been observed in patients with genotype 1 chronic hepatitis C and high insulin resistance as compared to patients with low insulin resistance [14].

Another question is whether the insulin resistance variations during treatment are the result of a selective direct antiviral effect (i.e., on insulin signal at the hepatocyte level) or rather an indirect consequence of antiviral treatment on combined metabolic factors (i.e., body mass index or adipokine variations during treatment). It has been observed that, in patients with HCV genotype 1 infections, therapy with peginterferon and ribavirin is associated with a decrease of body weight and insulin resistance [15]. Elevated leptin levels have been reported in patients with chronic HCV infection, and especially in those with metabolic risk factors, but it seems to be directly related to the degree of steatosis and of fibrosis [16]. These data support the hypothesis of a metabolic-related effect of antiviral therapy. However, a leptin level reduction without BMI modification during antiviral treatment in HCV patients has been reported [17], although there is no conclusive evidence to answer the former issue.

With this in mind, the aim of our study was to evaluate the relationship between insulin resistance and viral load modifications as well as the possible influence of body mass index (BMI) and serum leptin as metabolic confounders on insulin resistance kinetics in nondiabetic and noncirrhotic genotype 1 chronic hepatitis C patients during peginterferon alpha and ribavirin treatment.

## 2. Patients and Methods

The current study was designed to analyse data from 75 consecutive, nondiabetic patients with genotype 1 chronic HCV without cirrhosis who had not previously been treated with interferon alpha and/or ribavirin and who were recruited from two referral liver units (University of Genoa and San Paolo Hospital, Savona, Italy).

Seventy-five consecutive, nondiabetic, and noncirrhotic patients with genotype 1 chronic HCV who had not previously been treated with interferon alpha and/or ribavirin were included in this study. There were 45 men (60%) and 30 women (40%), with median age of 49 years (range 26–67). All patients had HCV-RNA levels higher than 1000 IU/mL, increased serum alanine aminotransferase (ALT) levels at screening, and a liver biopsy specimen taken in the 18 months prior to study entry showing chronic hepatitis. Patients were excluded if they had HCV genotype other than type 1 infection (i.e., HCV types 2–6), biopsy-proven cirrhosis, other causes of liver disease, hepatitis B virus infection, human immunodeficiency virus infection, autoimmune disorders, clinically significant cardiac or cardiovascular abnormalities, organ grafts, systemic infections, clinically significant bleeding disorders, evidence of malignant neoplastic diseases, concomitant immunosuppressive medication, fasting glucose levels >7 mmol/L or glucose levels >11.1 mmol/L at 2 hours after an oral intake of 75 grams of glucose (oral glucose tolerance test) or glycosylated hemoglobin > 48 mmol/mol (6.5%) or antidiabetic treatment, or any alcohol intake or drug abuse within the six months prior to study entry. Patients with a BMI ≥ 30 were also excluded from the study.

All 75 patients were treated with the same initial schedule of peginterferon alpha-2a (Pegasys, Roche Corp. at a dose of 180 *μ*g/kg/week and ribavirin (Copegus, Roche Corp.)) either 1000 mg/day (body weight < 75 kg) or 1200 mg/day (body weight > 75 kg). During treatment, patients were evaluated at monthly intervals (i.e., blood cell count, hemoglobin levels, platelets, thyroid function, and serum liver tests) to monitor compliance and side effects. Patients who showed a <2log_10_ drop in viral load at week 12 as compared to baseline or those whose HCV-RNA level was positive at week 24 discontinued treatment on the basis of the international guidelines. Conversely, patients who had a ≥2log_10_ drop in viral load at week 12 as compared to baseline and whose HCV-RNA level was negative at week 24 continued the treatment schedule for a total of 48 weeks. The study protocol was approved by the local Ethics Committees and patients gave written informed consent to participate.

### 2.1. Virologic Assessment and Definition of On-Treatment Response

All subjects were reactive to anti-HCV antibodies using a third-generation enzyme immunoassay EIA (Abbott HCV 3.0 Elisa). Serum HCV-RNA levels were quantified in all patients at the beginning of treatment and then at weeks 4, 12, 24, 48, and 72 by using a real-time polymerase chain reaction (PCR) (COBAS TaqMan). HCV genotype was determined using the INNO-LiPA HCV II kit (Bayer Diagnostics, Emeryville, CA).

Patients who had a ≥2log_10_ drop in viral load at week 12 as compared to baseline and whose HCV-RNA level was negative at week 24 were defined as early virologic responders (EVR). All EVRs also had end-of-treatment response (EOTR) defined as having negative HCV-RNA levels at week 48. Sustained virological response was defined as undetectable HCV-RNA levels in the serum at week 72 (i.e., 24 weeks after the end of treatment, which was also the end of follow-up). Patients whose viral load declined more slowly (<2log_10_ drop at week 12 as compared to baseline), those whose viral load dropped >2log_10_ at week 12 as compared to baseline and who still had positive HCV-RNA at week 24, those who became HCV-RNA positive after negativization before the end of treatment (breakthrough response), and those who became HCV-RNA positive after negativization at the end of treatment (relapsers) were all considered non-SVRs.

### 2.2. Liver Histology

All patients underwent liver biopsy within the 18 months prior to treatment. Paraffin embedded biopsies were analyzed by a single pathologist unaware of the clinical and biological data, except for the presence of chronic hepatitis C. This analysis was performed after hematoxylin-phloxin-safran, Perls, and picrosirius red staining. Liver biopsy specimens no less than 15 mm long or the presence of at least 10 complete portal tracts were required. Necroinflammatory activity was graded and fibrosis was staged according to Ishak's scoring system [18]. Steatosis was graded according to the percentage of hepatocytes containing cytoplasmic vacuoles. It was demonstrated as >30% in 4 patients, between >5% and <30% in 7 patients and <5% in the remaining 64 patients.

### 2.3. Clinical and Laboratory Determinations

Age, sex, and body mass index (BMI, calculated as weight in kilograms/height in square meters) were recorded for all patients at baseline, while serum levels of ALT, aspartate aminotransferase (AST), gamma-glutamyl transpeptidase (GGT), glucose (glucose HK UV enzymatic test [Olympus]), insulin (measured by a two-site immunoenzymometric assay (ST-AIA-PACK IRI, Tosoh Corporation, Tokyo, Japan)), adoponectin, and leptin (commercial radioimmunoassay, DRG diagnostics, Germany) were all measured after overnight fasting. Glucose levels were also determined at 2 hours after an oral glucose intake of 75 grams (oral glucose tolerance test).

Insulin resistance was determined by the homeostasis model assessment (HOMA) method using the following equation: HOMA-IR = fasting insulin (mIU/L) × fasting glucose (mmol/L)/22.5 [19]. HOMA-IR has been validated in comparison with the euglycemic/hyperinsulinemic clamp technique as the standard reference in both diabetic and nondiabetic patients [20]. HOMA-IR, serum leptin, and BMI were measured in all patients at baseline and at weeks 12 and 48.

### 2.4. Statistical Analysis

Database management and statistical analyses were performed using a statistical software package (SPSS 10.0.5). Univariate analysis was carried out on metabolic variables for their possible association with SVR. *t*-test or Pearson's correlation test (for continuous variables) and *X* ^2^ test (for categorical variables) were used. Pearson's correlation test was also used to assess the association among metabolic variables. Logistic regression was carried out for multivariate analysis and for the definition of relative risk of single variables. A *P* value less than 0.05 was considered statistically significant.

## 3. Results

Of 75 patients (45 males and 30 females), median age 48 years (range 26–67), 26 experienced SVR (34.7%).  Table 1 shows some baseline metabolic parameters in the whole population and the difference on the basis of gender. BMI was high in men, whereas leptin and adiponectin were significantly higher in women than in men. No variation of HOMA-IR was seen with regard to gender.

When we evaluated the influence of each variable against each one of the others, we found a significant association between HOMA-IR and both BMI (*P* = 0.010) and leptin (*P* = 0.000), but not between HOMA-IR and age (*P* = 0.59) or baseline adiponectin (*P* = 0.53). The same associations are shown in Table 2 as the relationship of baseline BMI, adiponectin, and leptin based on quartiles distribution.

Table 3 shows the association between baseline pretreatment variables and SVR. Only age and BMI, but not HOMA-IR, were significantly associated with SVR. Both age and BMI were also independently associated with SVR at multivariate analysis (OR 1.26; 95% CI 0.99–1.60; *P* = 0.051 and OR 1.05; 95% CI 1–00–1.11; *P* = 0.041, resp.), although the significance of age is weak.

We also assessed the quartile distribution of HOMA-IR, assuming the 75th percentile of HOMA-IR value, equal to 3.20, as the cut-off value of insulin resistance in our population. The patients in the top quartile of HOMA-IR distribution values who were classified as having insulin resistance showed no difference in SVR rates (36.8%) compared with patients in the other three quartiles of HOMA-IR (33.9%; 95% CI 0.29–2.60; *P* = 0.81).

### 3.1. Variations in Metabolic Parameters during Treatment

As reported in Table 3, baseline HOMA-IR was not significantly different in patients who achieved SVR compared to those who did not achieve SVR. However, when we assessed HOMA-IR variations during treatment on the basis of different kinetics of viral load decay, we found a significant reduction of HOMA-IR values at week 12 and a still but weakly significant reduction at week 48 from baseline in the EVR (*P* = 0.033 and 0.048, resp.) but not in the non-EVR groups (Table 4).

We also analyse the variations of BMI and serum leptin during treatment; two variable sproved to be significantly associated with HOMA-IR. However, no significant variations of both variables at weeks 12 and 48 were observed compared to baseline in the EVR versus non-EVR groups (Table 4).

## 4. Discussion

Chronic hepatitis C virus infection is now considered a metabolic disease due to its complex interaction with glucose metabolism, leading to insulin resistance (IR) and diabetes. Insulin resistance occurs very early in transgenic mice expressing the HCV core protein (8), and this is similarly observed in humans [21]. HCV is able to interfere with the insulin signaling pathway by downregulating the insulin receptor substrates 1 and 2 (IRS1/2) gene expression in liver tissue [22]. On the other hand, there is robust evidence that viral clearance improves insulin resistance in patients with chronic hepatitis C with genotype 1 [12], which clearly represents* in vivo* demonstration of genotype 1 HCV-induced insulin resistance.

Since 2005, low SVR rates in patients with chronic hepatitis C and insulin resistance (defined as HOMA-IR ≥ 2) have been reported, and two meta-analyses reinforced this observation [23, 24]. However, some considerations have to be made: many of the studies that are included in these meta-analyses are heterogeneous and include varying proportions of patients with metabolic risk factors. In these studies, different and often arbitrary cut-off values of HOMA-IR were reported. Furthermore, as outlined by Eslam and colleagues, colinearity between HOMA-IR and other metabolic variables and the characteristics of the patients may represent strong confounders when analysing data [24]. Regarding the characteristics of the patients, liver fibrosis is crucial in negatively influencing SVR. However, insulin resistance is directly involved in fibrogenesis and it is associated with more severe fibrosis in genotype 1 patients [10]. Thus, liver fibrosis is another confounding factor contributing to lower SVR rates in patients with high HOMA-IR [23].

In our study, however, HOMA-IR was not associated with SVR. One reason could be related to patient selection. Our cohort did not include patients with advanced fibrosis nor those with high HOMA-IR values (median 1.9) or high BMI (median 23.6), a feature that is in line with the observation by Eslam and colleagues. In fact, of all the studies in their meta-analysis which failed to find an association between insulin resistance and SVR, the mean baseline HOMA-IR was <3 and the prevalence of advanced fibrosis or cirrhosis was also low or even absent [24]. This observation supports the idea that HOMA-IR is predictive of response to antiviral treatment only in patients with metabolic risk factors or advanced fibrosis, whereas in those without metabolic risk factors or with lower fibrosis and in whom insulin resistance is mainly HCV-related, the HOMA-IR index is improved by antiviral treatment and has no impact on response [23]. A study of Fattovich and colleagues [25] seems to further confirm this hypothesis. In this study, insulin resistance, regardless of the method used to measure it, failed to predict viral response in patients with chronic hepatitis C without metabolic syndrome.

When we evaluated the difference in HOMA-IR values at baseline, at week 12 and at the end of treatment on the basis of viral kinetics, we observed a rapid decrease (week 12) of HOMA-IR value followed by a slight increase at the end of treatment (week 48) only in the group of patients with early viral abatement (EVR). This observation suggests an imbalance of the metabolic pathway in the early phase of treatment in patients who are unable to suppress viral replication. Many studies seem to support this hypothesis. Bortoletto and colleagues demonstrated that insulin resistance exerts its negative influence on viral decay very early, already in the first 24-hours after starting antiviral treatment [13]. On the other hand, high HOMA-IR values have been associated with low rates of rapid viral response (RVR) in genotypes 1 [26], 3 [27] and 4 [28].

The possible link between HCV infection, insulin resistance, and the early phase of viral kinetics during antiviral treatment involves the downregulation of IRS1/2 and upregulation of the suppressor of cytokine signaling-3 (SOCS-3) genes in liver tissue [9, 22]. SOCS-3 is a gene involved in the interferon signaling pathway and it is associated with poorer treatment outcome in patients with chronic hepatitis C viral genotype 1. Its overexpression is also associated with metabolic syndrome [29]. In this view, it could be speculated that, in patients with genotype 1-chronic hepatitis C without metabolic risk factors and in those whom we assume there is an HCV-related insulin resistance, HOMA-IR may be considered a surrogate marker of genotype 1b-induced upregulation of the SOCS3 gene in nonresponders to antiviral treatment.

Many studies have tried to demonstrate the beneficial effect of adding insulin sensitiser agents to peginterferon and ribavirin in order to enhance viral clearance in genotype 1 chronic hepatitis C with insulin resistance. Neither metformin nor pioglitazone proved the validity of this therapeutic strategy in the attempt to achieve SVR, although higher early viral response (at 4 and 12 weeks) was observed in the insulin sensitizer plus peginterferon and ribavirin groups compared with the control groups (peginterferon plus ribavirin) [30–32]. The results of these studies confirm that insulin resistance in genotype 1-HCV patients is the result of a more complex pathogenetic interrelation that is only marginally influenced by the activation of the proliferator-activated receptor-*γ* (PPAR-*γ*) pathway or via the metformin-induced hepatic AMP-activated protein kinase.

One of the goals of this study was to explore the association of some metabolic variables with insulin resistance. First, we found a different association with gender among various metabolic variables. No differences in HOMA-IR were observed between males and females, whereas BMI was higher in males. Although we expected higher leptin levels in males based on BMI, we found higher levels of both leptin and adiponectin in females. This observation was already reported by Lo Iacono and colleagues [17] and was found to be related to a higher grade of steatosis. Although this data was not assessed in our population due to a low prevalence of significant steatosis (only 11 out of 75 patients had steatosis in more than 5% of hepatocytes) a slight increase of serum leptin in female with higher degree of steatosis was observed.

Furthermore, our study is aimed at exploring whether HOMA-IR variations during antiviral treatment are associated with similar variations in leptin and BMI or not. Although we assessed both leptin and adiponectin at baseline, we chose to compare only leptin variations during treatment because only leptin and BMI, but not adiponectin, were associated with HOMA-IR.

Leptin is a 16-kDa polypeptide that is synthesized by mature adipocytes, skeletal muscle cells, and culture-activated hepatic stellate cells. Leptin expression is regulated by IL-1, TNF-*α*, and insulin, whereas its secretion, a prerogative of adipocytes alone, is proportional to the fat mass and provides antiobesity signals. Nonetheless, obese patients have elevated levels of leptin, suggesting that they are resistant to its action. One of the mechanisms of leptin resistance is an increase in SOCS3 levels, which impairs postreceptor signaling and leads to reduced adenosine monophosphate-activated protein kinase (AMPK) activation [33].

In our study, we observed no significant variations in either BMI or in leptin levels between baseline and weeks 12 and 48, unlike HOMA-IR. This issue seems to further suggest a selective effect of treatment on HCV-related insulin resistance. More information needs to be obtained by comparing insulin resistance to leptin levels during long-term follow-up after treatment.

Furthermore, unlike what was reported by Conjeevaram and colleagues [15], we did not observe a similar variation of HOMA-IR and BMI during treatment. However, our study population is not comparable to that of Conjeevaram et al.'s study in which BMI was above 25 in more than 77% of patients.

In summary, in our selected population of noncirrhotic, nonobese, and nondiabetic HCV chronic patients, HOMA-IR, as expression of insulin resistance, is not a predictor of SVR but rather a dynamic parameter influenced by viral load. In fact, its significant reduction in parallel with a consistent abatement of viral load during antiviral treatment (EVR patients) compared with patients who did not achieve significant drop in viral load (non-EVR) suggests that the viral load has a role in determining a state of “inflammatory” insulin resistance, irrespective of BMI and leptin levels.

In conclusion, our data support the hypothesis of a viral-related insulin resistance in chronic hepatitis C genotype 1 patients without a metabolic phenotype.

## Figures and Tables

**Table 1:** Baseline metabolic parameters of 75 patients with genotype 1 chronic hepatitis C on the basis of gender.

	All patients	Male	Female	OR 95% CI	*P*
Number of patients	75	45	30		

Age (median; range)	49 (26–67)	48 (26–67)	49 (31–67)	1.01 (0.97–1.05)	0.58
BMI (median; range)	23.6 (19.4–29.6)	24.1 (21.3–29.6)	22.5 (19.4–28.7)	0.82 (0.68–0.99)	**0.047**
Fasting glucose (median; range)	5.03 (2.20–6.88)	5.10 (2.41–6.78)	4.83 (2.21–6.52)	0.82 (0.54–1.26)	0.37
Fasting insulin (median; range)	8.84 (1.4–27.9)	8.51 (1.40–21.3)	9.1 (2.0–33.1)	1.02 (0.97–1.08)	0.40
HOMA-IR (median; range)	1.9 (0.3–10.5)	1.9 (0.3–10.3)	2.05 (0.3–10.5)	1.09 (0.89–1.34)	0.42
Leptin (median; range)	9.2 (2.2–55)	6.3 (2.2–29.2)	15.4 (3–55)	1.14 (1.06–1.23)	**<0.000**
Adiponectin (median; range)	13.5 (4.5–36.1)	12.2 (4.5–23.3)	17.05 (7.1–36.1)	1.16 (1.05–1.23)	**0.002**

**Table 2:** Mean baseline BMI, adiponectin, and leptin levels based on quartiles distribution of HOMA-IR.

HOMA-IR quartiles	1°	2°	3°	4°
	0.3–1.0	1.1–1.8	1.9–3.2	3.3–10.5
BMI	22.6	23.5	25.0	25.1
Leptin	7.0	10.5	13.9	20.7
Adiponectin	14.9	14.4	16.4	16.0

**Table 3:** Baseline pretreatment variables based on treatment outcome (SVR) at univariate and multivariate analyses.

	Univariate analysis				Multivariate analysis		
	SVR	Non-SVR	OR 95% CI	*P*	OR 95% CI		*P*
Number of patients	26	49					

Age (median; range)	48 (26–67)	49 (31–67)	1.07 (1.02–1.12)	**0.008**	1.26 (0.99–1.60)		**0.051**
Sex (M/F)	16/10	29/20	0.91 (0.34–2.4)	0.84			
BMI (median; range)	22.7 (19.4–29.1)	24.2 (21.3–29.6)	1.33 (1.07–1.66)	**0.01**	1.05 (1.00–1.11)		**0.041**
Fasting glucose (median; range)	4.86 (2.41–6.23)	5.1 (2.2–6.88)	1.16 (0.76–1.79)	0.49			
Fasting insulin (median; range)	8.0 (1.41–24.1)	8.9 (2.90–33.1)	0.98 (0.93–1.04)	0.58			
HOMA-IR (median; range)	1.93 (0.3–10.3)	2.25 (0.3–10.5)	0.95 (0.77–1.18)	0.65			
Leptin (median; range)	6.3 (2.2–29.2)	15.4 (3–55)	1.01 (0.96–1.05)	0.89			
Adiponectin (median; range)	12.2 (4.5–23.3)	17.05 (7.1–36.1)	1.02 (0.94–1.10)	0.67			

**Table 4:** On treatment HOMA-IR, BMI, and serum leptin variations based on early viral decay (EVR versus non-EVR).

	EVR (*n* = 36)					Non-EVR (*n* = 39)				
	Baseline		12 weeks		48 weeks	Baseline		12 weeks		48 weeks
HOMA-IR (median)	2.03 (0.3–5.6)	§	1.41 (0.33–3.35)	*ψ*	1.60 (0.42–4.18)	2.30 (0.4–10.5)	∧	2.18 (0.45–10.3)	∧	2.21 (0.81–9.23)
BMI (median)	22.9 (19.4–29.1)	∧	21.6 (19.4–29)	∧	21.9 (20–29.1)	24 (21.5–29.6)	∧	22.7 (20.3–29.1)	∧	22.6 (19.6–29)
Serum leptin (median)	10.5 (3.8–27.9)	∧	8.91 (3.3–39.6)	∧	12.6 (2.6–35.7)∖	10.15 (3–25.6)	∧	9.05 (3.8–25.7)	∧	9.70 (3.2–23.2)

^§^(12 weeks versus baseline): *P* = 0.033; ^*ψ*^(48 weeks versus baseline): *P* = 0.048; ^∧^(12 weeks versus baseline and 48 weeks versus baseline): *P* = NS. EVR: early viral response.
